# Intra Vitam Diagnosis of Neglected *Gurltia paralysans* Infections in Domestic Cats *(Felis catus)* by a Commercial Serology Test for Canine Angiostrongylosis and Insights into Clinical and Histopathological Findings—Four-Case Report

**DOI:** 10.3390/pathogens9110921

**Published:** 2020-11-06

**Authors:** Marcelo Gómez, Catalina García, Isabel Maldonado, Nikola Pantchev, Anja Taubert, Carlos Hermosilla, Manuel Moroni, Pamela Muñoz, Alejandra Duran, Marcelo Mieres, Javier Ojeda

**Affiliations:** 1 Instituto de Farmacología y Morfofisiología, Facultad de Ciencias Veterinarias, Universidad Austral de Chile, Campus Isla Teja, Valdivia 5090000, Chile; catalina.garcia@alumnos.uach.cl (C.G.); isabel.maldonado@uach.cl (I.M.); 2 IDEXX Laboratories, 70806 Kornwestheim, Germany; nikola-pantchev@idexx.com; 3 Institute of Parasitology, Justus Liebig University Giessen, 35392 Giessen, Germany; anja.taubert@vetmed.uni-giessen.de; 4 Instituto de Patología Animal, Facultad de Ciencias Veterinarias, Universidad Austral de Chile, Campus Isla Teja, Valdivia 5090000, Chile; manuelmoroni@uach.cl (M.M.); pamela.munoz@uach.cl (P.M.); 5 Instituto de Ciencias Clínicas Veterinarias, Facultad de Ciencias Veterinarias, Universidad Austral de Chile, Campus Isla Teja, Valdivia 5090000, Chile; alejaduran08@gmail.com (A.D.); mmieres@uach.cl (M.M.); javierojeda@uach.cl (J.O.)

**Keywords:** *Gurltia paralysans*, gurltiosis, feline, diagnosis

## Abstract

*Gurltia paralysans* is a metastrongyloid nematode which belongs to the Angiostrongylidae family and presents tropism for veins of the subarachnoid space in vivo of domestic and wild felids causing a progressive and chronic clinical manifestation of paraparesis/paraplegia. The geographic distribution of *G. paralysans* includes rural and periurban areas of South America and was recently reported in Europe. To date, a definitive diagnosis of feline gurltiosis is still conducted by post-mortem examination of the spinal cord in affected animals. A presumptive diagnosis of feline gurltiosis can also be achieved based on data of compatible clinical signs and associated epidemiological risk factors. The aim of this preliminary study was to evaluate the commercial serological test Angio Detect TM^®^ (IDEXX Laboratories) as a possible diagnostic method of feline gurltiosis in vivo. For the study, 10 domestic felines (*Felis catus*) which originated from a high endemic area of Southern Chile, were analyzed. All felines presented chronic paraparesis or severe paraplegia. Subsequently, commercial Angio Detect TM^®^ serological tests for the detection of closely related *Angiostrongylus vasorum* in canids were performed according to manufacturer’s instructions. Conducted serological tests were positive in seven of ten felines showing paraplegia/paraparesis and presumably infected with *G. paralysans*, and four of them were additionally necropsied, and presented macroscopic findings compatible with feline gurltiosis. Furthermore, the presence of adult female and male *G. paralysans* specimens at the level of the subarachnoid vasculature in affected spinal cord segments were observed during necropsy. Histopathology demonstrated severe eosinophilic meningomyelitis, coagulopathies with thrombosis in *G. paralysans*-parasitized leptomeningeal veins. Results of this preliminary study suggest a cross-reaction between *A. vasorum*-specific antigens, which also parasitize blood vessels in vivo, and *G. paralysans* when using an Angio Detect TM^®^ test, which suggests its helpful use as a new diagnostic method for feline gurltiosis in live domestic felines. Additional specific antigen research will be required in order to better understand the sensitivity and specificity of *A. vasorum* antigens used in this test and for existing cross-reactivity with *G. paralysans-*derived antigens for future a suitable intra vitam immunodiagnosis of neglected feline gurltiosis.

## 1. Introduction

**Figure 1:**
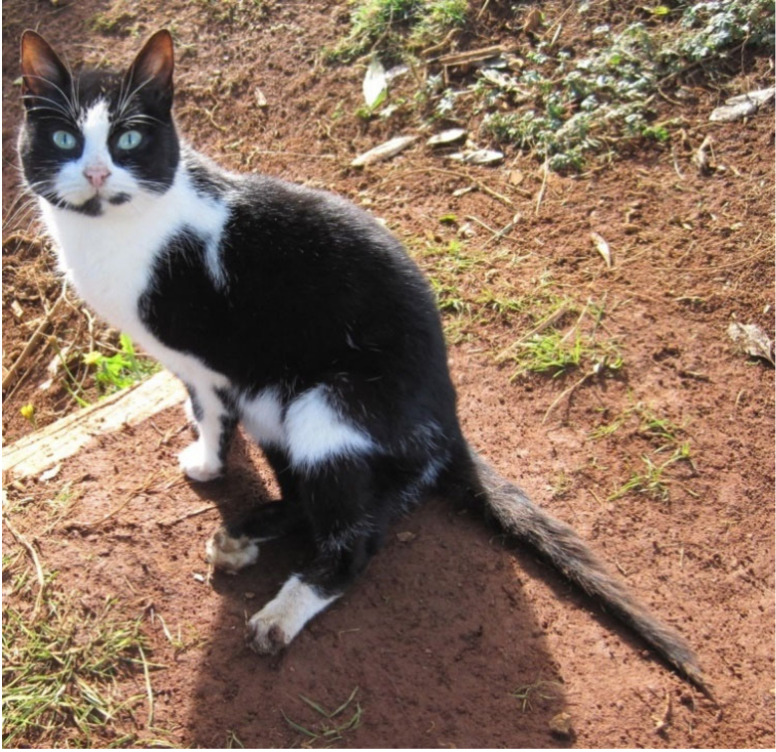
A domestic cat (*Felis catus*) with paraparesis due to *Gurltia paralysans* infection.

*Gurltia paralysans* (Nematoda: Order Strongylida; Family Angiostrongylidae) is a metastrongyloid parasite which causes chronic meningomyelitis in domestic cats (*Felis catus*) and some wild felids from the genus *Leopardus* such as the kodkod or guigna (*Leopardus guigna*) and margay (*Leopardus triginus*) [1,2,3]. Its geographic distribution includes areas of Chile, Argentina, Uruguay, Colombia, and Brazil and recently Tenerife Island, Spain [2,4,5,6,7,8]. The nematode mainly dwells within feline leptomeningeal veins and parenchyma of the spinal cord, producing progressive paraparesis, paraplegia, fecal or urinary incontinence, and/or tail paralysis [1,2,9] (Figure 1). The life cycle of the parasite is still unknown, but it is probably heteroxenous, as for other closely related metastrongyloid nematodes [9,10].

Imaging studies (computed tomography (CT), myelography, and magnetic resonance imaging (MRI)) indicate that *G. paralysans* infections induce lesions in the thoracolumbar, lumbar, or sacral regions, suggesting diffuse inflammatory spinal cord lesions resulting in severe myelitis [9] or meningomyelitis [1]. While chronic myelopathy signs are frequently associated with feline gurltiosis, a recent case of ectopic nematode localization, namely in the anterior chamber of the eye resulting in ocular feline gurltiosis form, was recently reported [8]. Necropsy findings in *G. paralysans*-infected domestic cats (*F. catus*) and wild felids include diffuse submeningeal congestion of lumbar, sacral, coccygeal spinal cord segments, and presence of several intravascular adult nematodes, larvae and pre-adult stages have been identified histologically in the meningeal veins of the spinal cord, with subsequent congestion, thrombosis, and thickening of subarachnoidal vessels [1,2,7,9].

**Figure 2:**
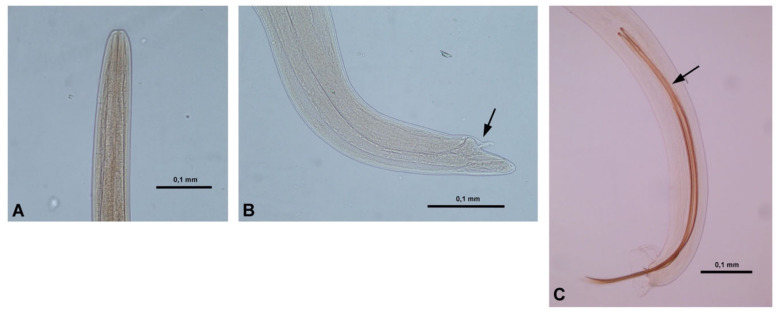
Morphological characteristic for identification of *Gurltia paralysans*. Anterior end of an adult female specimen (**A**). The cephalic region is slender and 0.032–0.036 mm wide and with absence of cervical papillae. Posterior end of a female specimen (**B**). Females have a curved end and a vulvar aperture with a folded flap (arrow) and the vulva opens 0.10–0.11 mm from tail tip. Posterior end of an adult male specimen (**C**). Males have a curved end with a copulatory bursa and long curved spicules (arrow) of about 0.72–0.82 mm length.

The definitive diagnosis of feline gurltiosis can only be performed with a post-mortem examination by demonstrating and identifying, morphologically, nematodes in the spinal cord vasculature [1,9] (Figure 2). An exhaustive clinical exam and complementary tests (cerebrospinal fluid (CSF), hemogram, fecal examination), and imaging findings are necessary to exclude other myelopathies in order to establish a presumptive intra vitam diagnosis of feline gurltiosis [9,11]. Consistently, we have recently sequenced the partial 18S rRNA gene of *G. paralysans* (GenBank: JX975484) and used it to run a phylogenetic analysis of *G. paralysans*, revealing nucleotide homology scores of 97% with *Angiostrongylus vasorum* [10]. *A. vasorum* is another metastrongyloid nematode which inhabits the right ventricle and pulmonary vasculature in domestic dogs (*Canis familiaris*), wolves *(Canis lupus)* [12], golden jackals *(Canis vulpes)* [13] and foxes *(Vulpes vulpes)* [14] producing respiratory distress, disseminated intravascular coagulopathy and/or CNS hemorrhages [15]. Recently, a natural infection of *A. vasorum* of in a domestic cat (*Felis catus*) was reported [16]. A rapid diagnosis of canine angiostrongylosis can be obtained by the use of the Angio Detect TM^®^ (IDEXX laboratories Inc.) serological test, which detects circulating antigens produced by *A. vasorum* [17,18]. Thus, the objective of this work was to evaluate the cross-reactivity of *G. paralysans-*derived antigens with *A. vasorum-*specific antigens using this commercial serologic test (Angio Detect TM^®^) developed for the diagnosis of canine angiostrongylosis in domestic dogs and the possibility of its use in domestic cats with a suspected clinical diagnosis of feline gurltiosis.

## 2. Results

**Figure 3:**
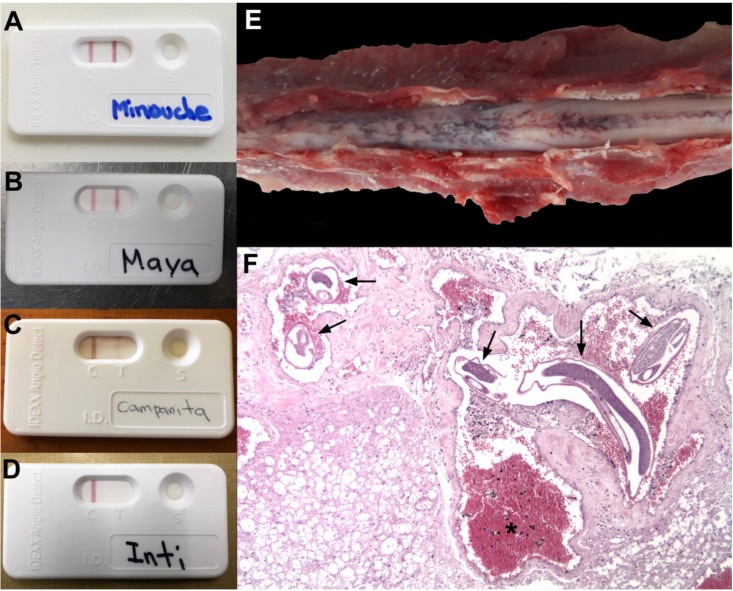
Serological results of Angio Detect TM^®^ (IDEXX Laboratories) in domestic cats affected with suspected feline gurltiosis. (**A**) Coloration intensity in intensive reactions (+++), good reactions (++) in (**B**), slight reactions (+) in (**C**) and negative reactions (−) in (**D**). Macroscopic spinal cord lesions in an affected domestic cat with feline gurltiosis showing congestive submeningeal veins (**E**). Histopathological findings of the feline spinal cord showing sections of adult *Gurltia paralysans* nematodes ( ) and congestive vasculature inside the subarachnoid space (*) (HE, 10X) (**F**).

From the 10 examined domestic cats, seven were identified as positive from the serological examination using the Angio Detect TM^®^ Test (IDEXX Laboratories; Table 1). From these, three showed intensive coloration (+++) on the test, one showed good visible coloration (++), and finally three showed slight coloration (+), and all the control lines of the applied test were positive (Figure 3A–D). Out of the seven positive cats, a complete necropsy study was performed in four of them where severe macroscopic lesions compatible with feline gurltiosis were found. Additionally, in these four animals, adult female/male nematodes morphologically identified as *G. paralysans* were unveiled (Table 1). Simultaneously, three of the positive domestic cats were positive for *Aelurostrongylus abstrusus* via Baermann funnel analysis.

Neurological examination findings included paraparesis (*n* = 10), symmetric pelvic limb ataxia (*n* = 7), asymmetric pelvic limb ataxia (*n* = 3), tail paralysis (*n* = 8), plantigrade stance (*n* = 2), pelvic limb proprioceptive deficit (*n* = 10), pelvic limb muscle atrophy (*n* = 2), urinary incontinence (*n* = 3), fecal incontinence (*n* = 1), perianal hyperesthesia (*n* = 4), hindlimbs hyperesthesia (*n* = 1) increased patellar reflex (*n* = 3), decreased patellar reflex (*n* = 2), cross-extensor reflex (*n* = 3), positive Babinsky reflex (*n* = 1) and lumbosacral hyperesthesia (*n* = 4) (Table 2). The neuroanatomic localization of the affected domestic cats was associated with involvement of thoracolumbar (T3-L3) and/or lumbosacral (L6-Cd4) spinal cord segments (please refer to and as Supplementary Data).

A microdissection and visual examination of the spinal cord after meningeal incision in all necropsied cats revealed characteristic vascular leptomeningeal lesions associated with *G. paralysans* infection. Nematodes were extracted from all affected spinal cord vessels and microscopically identified through the morphoanatomical and morphometric features of *G. paralysans,* as previously described elsewhere [2,10]. From the necropsied animals, 12 adult of *G. paralysans* (10 females and 2 males) were recovered from the subarachnoid vasculature. Necropsy findings included congestion of vasculature in the lumbar subarachnoid space, tortuous leptomeningeal veins, section of adult nematodes and thrombus inside meningeal vein vasculature (Figure 3E). *G. paralysans*-parasitized tissue sections of thoracolumbar/lumbosacral spinal cord segments revealed severe phlebitis of leptomeningeal veins thereby showing disseminated thrombosis, intra- and extravascular hemorrhages, and granuloma formation, mainly composed of eosinophils, monocytes and polymorphonuclear neutrophils (PMNs) around the affected veins and edema of spinal cord parenchyma. Some of these granulomas and/or thrombi contained *G. paralysans* eggs, pre-adult and/or adult stages resulting in severe chronic myelitis and/or meningomyelitis (Figure 3F). Neither macroscopic lesions in visceral thoracic nor abdominal organs were observed.

In our study, 7/10 felines with a suspected diagnosis of feline gurltiosis tested positive in the serological examination using an Angio Detect TM^®^ Test (IDEXX Laboratories). From these seven positive cats, a complete necropsy was performed and in four of them post-mortem lesions compatible with feline gurltiosis, as well as nematodes morphologically identified as *G. paralysans,* were found.

## 3. Discussion

Intra vitam diagnosis of feline gurltliosis is still complicated and very challenging since *G. paralysans* larvae are neither detected in feces nor in body fluids. Alternatively, feline gurltiosis diagnosis can be suspected based on clinical signs, diagnostic imaging findings (i.e., CT, Magnetic Resonance Imaging (MRI), and epidemiological features [9]. A final etiological diagnosis is mainly obtained by post-mortem examinations of spinal cords with characteristic vascular lesions and identification of nematodes and/or eggs within extended leptomeningeal vessels [2,9,11].

Commonly reported presentations of feline gurltiosis include myelitis, usually with thoracolumbar and/or spinal cord segment involvement [1,2]. Usual neurologic manifestations in these cases include symmetric and asymmetric paraparesis/paraplegia, pelvic limb ataxia, spinal hyperesthesia, sphincter disturbances (urethral and anal), altered pelvic limb spinal reflexes and tail paralysis similar to signs observed in the confirmed feline gurltiosis cases of this study. Ocular migration and associated chorioretinitis and posterior synechiae have been reported in one case with *G. paralysans* infection; however, these was not observed in the present study [8]. It has been postulated that *G. paralysans* nematodes migrate into vessels through the retrograde vertebral venous plexus from abdominal veins connections to reach their specific anatomic sites of the spinal cord between the thoracolumbar (T3-L3), lumbosacral (L4-S3) and caudal segments producing the associated parasitic myelopathies [2,9].

Microscopic findings in four cases revealed vascular changes including spinal cord deformation, subarachnoid and parenchymal vein congestion, varicose veins, thrombus and presence of nematode sections compatible with *G. paralysans* infections. These findings are in accordance with previous reports that revealed parasitic vascular myelitis and intralesional adult parasites that affect mainly the subarachnoid space, the thoracolumbar and lumbosacral spinal cord segments [1,7,11].

The Angio Detect TM^®^ test (IDEXX Laboratories) is considered a rapid pet-side antigen blood test, which is specific for the detection of *A. vasorum* infections in canid species [17]. Unlike other testing methods, it is not compromised by the intermittent presence of *A. vasorum*-L1 in feces. This commercial test was found to have a sensitivity of 97.1% and a specificity of 98.9% in dogs [17]. Recent studies evaluating the Angio Detect TM^®^ Test (IDEXX Laboratories) have shown the serologic cross-reactivity of *A. vasorum* with *Angiostrongylus chabaudi* in European wildcats and *Angiostrongylus daskalovi* in free-ranging badgers [19]. Although, domestic and wild cats have been known to be clinically affected with *A. chabaudi*, thereby showing granulomatous pneumonia, parenchymal hemorrhages and alveolar emphysema [20]; a demonstration of *A. chabaudi* adults, larvae or associated leptomeningeal lesions have not been observed in feline angiostrongylosis in wild Chilean kodkods [21]. Three of the ten felines examined in this study were positive for infections with *A. abstrusus*, a metastrongyloid nematode that also belongs to the family Angiostrongylidae and reported to occur in domestic and wild cats with symptoms varying from subclinical to fatal respiratory diseases [22,23]. Although, the Baermann funnel technique is the most frequently used method for the diagnosis of metastrongyloid infections, this technique has failed so far to detect patent *G. paralysans* infections, particularly during periods when there is no larval removal independent of the existence of clinical signs [8,24]. As mentioned, the complete life cycle of *G. paralysans* to date is still unknown; however, it is presumed that it could share obligate intermediate hosts (terrestrial slugs/snails) and/or paratenic hosts (lizards, amphibians, birds, rodents) of other closely related metastrongyloid feline nematodes present in Chilean territories, such as *A. abstrusus* [2], *A. chabaudi* and *Troglostrongylus brevior* [21]. Consequently, frequent concomitant infections with *A abstrusus* might be suitable to assume, as demonstrated elsewhere [23], as is the possibility of antigenic cross-reactions with other metastrongyloids such as *A. vasorum*, *A. chabaudi*, *G. paralysans* and *T. brevior*, while there are no studies covering these issues [2]. However, in a recent study, 25 domestic cats tested positive, by the Baermann funnel technique, for *A. abstrusus*, 16 for *T. brevior* and 9 were positive for nematodes and scored negative in the Angio Detect TM^®^ Test (IDEXX Laboratories) for circulating *Angiostrongylus-*specific antigens [25]. Additionally, ELISA tested for the presence of *A. abstrusus* antibodies and showed absences of antigenic cross-reactions in sera from cats experimentally infected with other nematodes and tapeworms (*Toxocara cati*, *Ancylostoma tubaeforme* and cestodes [Taeniidae]) [26]. A recent new RT-PCR targeting the second internal transcribed region of ribosomal DNA (ITS2) of *A. vasorum* did not amplify a range of helminths including *A. abstrusus* [27]. Nonetheless, antigenic cross-reactivity has been observed between *A. vasorum* and *Crenosoma vulpis* and *A. vasorum* using ELISA tests, probably due to shared antigens/epitopes [17,28]. However, it seems that the Angio Detect TM^®^ test can show cross-reactivity only between species of the genus *Angiostrongylus* or very closely related species [19] such as *G. paralysans* [10]. Considering the close phylogenetic relationship between *A. vasorum* and other nematodes, it is most likely that many metastrongyloid proteins and/or antigens/epitopes share close structural homologies [29]. The similarity between 18S ribosomal RNA gene sequences of *G. paralysans* and *A. vasorum* are 97.5% and 97.05% with *A. chabaudi* [10], thereby reconfirming a close phylogenetic relationship. Thus, *A. vasorum-*derived antigen cross-reactivity with *G. paralysans* cannot be ruled out but needs further clarification as seven out of ten infected animals tested positive using this immunodiagnostic tool.

Schnyder et al. (2014) [17] postulated that the formation of antigen–antibody complexes could inhibit the detection of antigens, this being the explanation for both the Angio Detect TM^®^ Test and ELISA to give negative results for cases with a positive diagnosis of *A. vasorum* using the Baermann technique, as previously demonstrated with *Dirofilaria immitis.* This explanation might be extrapolated in the case of *G. paralysans* infections, being a plausible reason for the appearance of false negatives or lower intensity results in chronic infections. Additionally, the presence of other causes of myelopathy in negative domestic cats cannot be totally ruled out (e.g., spinal neoplasia, inflammatory/infectious conditions, traumatic lesions, vascular myelopathy, intervertebral disk herniation) [30]. Serological tests for *A. vasorum*-specific antibody detection based on adult-, excretory/secretory (ES) or L1 antigens showed a sensitivity of up to 85.7% and a specificity of 98.8% during pre-patency [29]. However, Schucan et al. (2012) [31] found cross-reactions using adult-somatic, adult ES- and L1-somatic antigens from the sera of dogs infected with *C. vulpis*, *D. immitis*, *D. repens* and *Eucoleus aerophilus*. Additional serological diagnostic research is required on *A. vasorum*-derived antigens which might representatively establish sensitivity and specificity for the existence of cross-reactivity with intra vitam circulating *G. paralysans-*specific antigens in affected cats.

Based on these preliminary findings, the Angio Detect TM^®^ Test (IDEXX Laboratories) can alternatively be used for effective and rapid intra vitam diagnosis for cats displaying clinical signs of chronic *G. paralysans* infections. Unambiguously, further research is required to evaluate cross-reactivity with other feline metastrongyloid species present in Chile, namely *T. brevior* and *A. chabaudi* [21]. The use of the Angio Detect TM^®^ Test is considered as a promising technique for use in feline medicine for identification of *Angiostrongylus* spp. infections combining the diagnostic approach with copro-microscopy analysis, and DNA-based assays which could minimize chances of false negative results [25].

Limitations of our study are firstly that it included a rather small sample size—only animals with severe neurological signs—and therefore perhaps is not truly representative the whole cat population exposed to this neglected and underdiagnosed parasite. Another limitation is that post-mortem analysis and identification of nematodes was only conducted in four of the suspected cases of feline gurltiosis. Additionally, the lack of *G. paralysans*-specific PCRs performed in the serum or CSF of sampled animals and complete post-mortem necropsies of all suspected animals as confirmation methods are further limitations. Nonetheless, we recently developed a novel semi-nested PCR diagnosis for the detection of *G. paralysans* DNA circulating in positive serum samples [32]. As feline angiostrongylosis and troglostrongylosis have recently been reported to occur in wild felids of Chile [21] future surveillance of domestic and endemic wild cat species (i.e., guigna (*L. guigna*), pampas cats (*Leopardus colocolo, Leopardus pajerus*) and puma (*Puma concolor*)) are necessary.

## 4. Materials and Methods

### 4.1. Case Definition and Selection

Domestic cats (*n* = 10) with neurological signs suggestive of feline gurltiosis and presented to the Veterinary Hospital, Faculty of Veterinary Sciences of the University Austral of Chile (UACh), Valdivia, Chile, were selected for evaluating a commercial Angio Detect TM^®^ Test (IDEXX Laboratories Inc., USA) to assess whether this immunological test allows cross-reaction with *G. paralysans-*specific antigens. Domestic cats included in the study originated from rural areas of the Los Rios region (33°24′S, 70°40′W), Southern Chile, where previous cases of *G. paralysans* infections have been diagnosed [2,9,10]. Cats were eligible as a suitable study case if they were adults (> one year of age), living in rural areas, and if they had, after a complete neurologic examination, signs compatible with feline gurltiosis including chronic signs (for >3 weeks) of paraplegia/paraparesis, pelvic limb ataxia, urinary/fecal incontinence, indoor/outdoor activities, or only outdoor lifestyle, and laboratory results included lower hematocrit (reference value 24–45%) or mean corpuscular hemoglobin concentrations (MCHbC; reference value 300–350 g/L), based on previously reported studies dealing with clinical manifested feline gurltiosis and/or associated biochemical blood parameters [9]. The owners were contacted and informed on the objectives of the study and kindly asked to voluntary donate their cats for this investigation. Cats with severe disease were euthanized with the consent of owners. We conducted this study in the Veterinary Hospital at the Faculty of Veterinary Sciences (UACh) in strict accordance with the recommendations of the Chilean National Commission of Sciences and Technologies, the guidelines of the Animal Care and Use Committee of the UACh, Valdivia, Chile, as well as in accordance with current Chilean Animal Protection Laws.

### 4.2. Cat Sera and Serologic Essay

Sera from suspected cases of feline gurltiosis were used for a prospective evaluation of a rapid device for serological detection of circulating *A. vasorum*-derived antigens in dogs (Angio Detect^TM^ Test, IDEXX Laboratories, France). This test is used for routine in-clinic diagnosis of canine angiostrongylosis. A whole blood sample (2 mL) was collected from the accessory cephalic vein in EDTA (ethylenediaminetetraacetic acid)-containing tubes (BD Vacutainer EDTA, Becton Dickinson, Lane Cove, NSW, Australia), which were centrifuged and the sera was preserved/frozen at −20 °C. Subsequently, each serum sample was examined using the Angio Detect TM^®^ Test according to the manufacturer’s instructions. All sera samples were tested with this commercial kit, a lateral flow immunochromatography assay, which includes a positive control field. Tests were performed by only one researcher following the manufacturer’s instruction and within the indicated expiry dates. Reading of the test was performed strictly at 15 min after reactions. Results were semi-quantitatively evaluated based on the color intensity (+ = slight but visible coloration, ++ = good visible coloration, +++ = intensive coloration), according to results previously reported [16]. Additionally, cats were also screened through a coprological examination through the Baermann funnel migration technique for the detection of various metastrongyloid first-stage larvae (L1).

### 4.3. Necropsies and Histological Tissue Sample Collection

A complete necropsy study, following a standardized protocol, was performed in the animals with the most severe clinical signs and previous owner´s consent. Spinal cord samples from affected areas were obtained from four examined cats. Samples were fixed in 10% buffered formalin and paraffin-embedded sections, stained with hematoxylin and eosin and microscopically analyzed for histopathological evaluation as described elsewhere [2].

## Figures and Tables

**Table 1:** Summary of the results on clinical signs duration, Baermann technique, serological Angio Detect TM^®^ test, number of *Gurltia paralysans* nematodes found in the spinal cord of the felines subjected to necropsy, and post-mortem findings of 10 felines with suspected feline gurltiosis.

ID	Gender	Neurologic Signs Duration	Baermann Test	Angio Detect TM^®^	Nº of Adult *G. paralysans*	Necropsy Findings
1	F	5 m	n/d	+++	6	Submeningeal congestion, tortuous veins
2	M	12 m	+	-	n/d	
3	F	2 m	-	+	1	Submeningeal congestion, tortuous veins
4	F	6 m	-	+	1	Submeningeal congestion, tortuous veins
5	F	36 m	-	+	n/d	
6	F	12 m	+	++	n/d	
7	F	36 m	-	-	n/d	
8	M	10 m	n/d	-	n/d	
9	F	12 m	+	+++	4	Submeningeal congestion, tortuous veins
10	F	4 m	-	+	n/d	

F = female; M = male; m = month; n/d = not done.

**Table 2:** Frequency of clinical findings observed in neurological examination performed on 10 felines with signs suggestive of feline gurltiosis.

Neurologic Signs	*n* =	ID (F)
Paraparesis	10	1, 2, 3, 4, 5, 6, 7, 8, 9, 10
Ataxia (hindlimbs)	10	Symmetric ataxia: 1, 2, 3, 6, 8, 9, 10 asymmetric ataxia: 4, 5, 7
Tail paralysis	8	mild: 2, 3, 5, 6, 8, 9complete: 4, 10
Plantigrade stance	2	2, 4
Proprioception deficit (hindlimbs)	10	1, 2, 3, 4, 5, 6, 7, 8, 9, 10
Muscular atrophy (hindlimbs)	2	3, 9
Urinary incontinence	3	3, 4, 10
Fecal incontinence	1	4
Lumbosacral hyperesthesia	4	4, 5, 8, 9
Perianal hyperesthesia	2	9, 10
Hindlimbs hyperesthesia	1	1
Patellar reflex	5	Increased: 6, 7, 10. Decreased: 8, 9
Cross-extensor reflex	3	7, 8, 10
Positive Babinski reflex	1	9

## Supplementary Materials

The following are available online at https://www.mdpi.com/2076-0817/9/11/921/s1, Video S1: domestic cat (*Felis catus*) with ambulatory paraparesis associated with *Gurltia paralysans* infection, Video S2: domestic cat (*Felis catus*) with paraplegia and tail paralysis.
